# Serum legumain is a potential biomarker for community-acquired pneumonia: a prospective cohort study

**DOI:** 10.7150/ijms.106118

**Published:** 2025-02-03

**Authors:** Xian-Ling Meng, Kai-Shu Ma, Kai-Xin Qu, Zhen-Yu Cheng, Lin Fu, Yi-Qing Qu

**Affiliations:** 1 Department of Pulmonary and Critical Care Medicine, Qilu Hospital of Shandong University, Jinan, Shandong, 250012, China.; 2 Department of Respiratory and Critical Care Medicine, Funan County People's Hospital, Fuyang, Anhui, 236300, China.; 3 Department of Respiratory and Critical Care Medicine, Second Affiliated Hospital of Anhui Medical University, Hefei, Anhui, 230601, China.; 4 Institute of Respiratory Diseases, Second Affiliated Hospital of Anhui Medical University, Hefei, Anhui, 230601, China.; 5 Center for Big Data and Population Health of IHM, The Second Affiliated Hospital of Anhui Medical University, Hefei, Anhui, 230601, China.

**Keywords:** Community-acquired pneumonia, Serum biomarker, Legumain, Severity, Prognosis

## Abstract

**Background:** Legumain is a cysteine endopeptidase that belongs to the C13 family. Many studies have revealed that legumain plays a vital pathogenic role in various respiratory diseases. The aim of this study was to explore the role of legumain in community-acquired pneumonia (CAP).

**Methods**: Serum samples were collected from 293 CAP patients on admission. The concentration of serum legumain was detected via an enzyme-linked immunosorbent assay. The relationship between serum legumain and CAP was assessed.

**Results**: Serum legumain concentrations were increased in severe CAP patients compared to the concentrations of mild CAP patients. The Spearman rank correlation coefficient suggested that the serum legumain concentration was strongly associated with many clinical indicators. Additionally, linear regression analysis revealed that the serum legumain concentration was positively correlated with the CURB-65, PSI, SMART-COP, and APACHE II scores. Moreover, the serum legumain concentration on admission was elevated in CAP patients who underwent mechanical ventilation, vasoactive agent therapy, ICU admission, and who died during hospitalization. CAP patients with higher serum legumain expression had poor prognostic outcomes. The predictive value of the serum legumain concentration for prognosis was similar to that of the severity score.

**Conclusions**: Serum legumain concentration is positively related to disease severity and a poor prognosis, indicating that serum legumain can be used as an indicator of disease severity and a prognostic indicator for CAP patients.

## Background

Pneumonia is a type of acute respiratory illness that affects the distal bronchial tree and alveoli of the lungs in humans. Pneumonia has become a significant factor influencing morbidity and mortality worldwide 1. Frequent categories of pneumonia include community-acquired pneumonia (CAP), which mainly occurs outside of hospital settings and is caused by microorganisms, and hospital-acquired pneumonia, which occurs more than 48 hours after hospitalization 2-3. CAP is a life-threatening disease that is accompanied by acute inflammation in the lung parenchyma 4. It is estimated that there are more than 5 million pneumonia patients in America and that one in five patients need to be hospitalised. Approximately 60000 people die from pneumonia or influenza worldwide every year 5. Moreover, children and the elderly are at high risk for CAP 6. An epidemiological study revealed that the incidence rate in adults in America is 5.16~6.11‰. However, the rate may be as high as, 12 to 18‰ in children younger than 4 years and 20‰ in individuals over the age of 60 7. Among these at-risk groups, partial cases may progress to severe CAP and severe acute respiratory distress, even resulting in death 8.

Legumain belongs to the C13 family of cysteine proteases and specifically hydrolyses asparaginyl bonds 9. In mammals, legumain was first identified in pigs as a glycoprotein. Mammalian legumain is localized mainly to chromosomes and is highly expressed in multiple organs 10, 11. Under normal conditions, prolegumain of 433 amino acids is synthesised to inactive zymogens and inhibit substrate proteolysis. Prolegumain is subsequently transferred from the Golgi apparatus to the endolysosomal compartments. Finally, prolegumain is cleaved to the active legumain in lysosomes 12. Previous investigations have demonstrated that legumain is involved in extracellular matrix degradation, cell proliferation, angiogenesis, and other physiological processes 13, 14. In addition, legumain deficiency alleviates acute tubular injury in mice by repressing ferroptosis 15. In contrast, legumain deficiency inhibits macrophage efferocytosis and leads to cardiomyocyte death 16. Moreover, increasing evidence indicates that legumain is involved in many pulmonary diseases. An animal experiment suggested that legumain elevation promotes pulmonary arterial hypertension in mice by aggravating collagen deposition 17. In addition, the plasma legumain concentration is increased in individuals with SARS-CoV-2 infection 18. Moreover, the expression of legumain is upregulated in the lungs of mice with bleomycin-induced pulmonary fibrosis 19, and legumain can promote fibrogenesis through activating transforming growth factor-β1 and enhancing the synthesis of the extracellular matrix 20. Nevertheless, the relationship between legumain and CAP is unclear.

Due to legumain's notable functions in various diseases, this study sought to analyse the relationships between serum legumain and severity and prognosis in CAP patients. Therefore, a prospective cohort study was designed and carried out. Eligible CAP patients were selected and recruited from hospitals. Biological samples and basic information were gathered. Serum legumain was measured in CAP patients. The relationship between serum legumain and CAP in patients was estimated by various statistical models. Our investigation initially provided important clues about the role of legumain in CAP patients.

## Materials and Methods

All CAP patients were enrolled from two hospitals, the Second Affiliated Hospital of Anhui Medical University and Funan County People's Hospital. All patients were diagnosed and complied with the CAP diagnostic criterion 21. Briefly, all cases occurred in the community and had obvious clinical characteristics of pneumonia and distinct chest diagnoses. In accordance with previous studies from our group, we established inclusion and exclusion criteria 22-25. Moreover, to eliminate patients with coronavirus disease 2019 (COVID-19), all enrolled subjects underwent nucleic acid testing, and patients with negative results were selected. Moreover, other concurrent infections were excluded. After hospitalization, peripheral fasting blood was collected from CAP patients before intervention or treatment. In addition, basic data, consisting of demographic characteristics, examination data, and chest radiograph information, were obtained from the medical records system. The degree of disease was evaluated by the scoring system, which mainly consisted of the CURB-65, PSI, SMART-COP, and APACHE II scores.

### Enzyme-linked immunosorbent assay (ELISA)

Serum samples were centrifuged and separated. Then, the serum samples were stored in an ultralow temperature freezer until the experiments. Human legumain ELISA kits (CSB-EL012903HU, https://www.cusabio.com/) were purchased from CUSABIO (Wuhan, China). The contents of serum legumain were determined via ELISA according to the manufacturer's instructions.

### Statistical analysis

In the present study, all the data were analysed via SPSS and GraphPad Prism software. The enumeration data are presented as frequencies. Additionally, the measurement data are expressed as the means or medians. Differences in the measurement data were explored by ANOVA or nonparametric tests with Bonferroni correction. The differences in enumeration information were evaluated via the χ^2^ test. Correlations between two indicators were analysed with the Spearman rank correlation coefficient. The relationships between the serum legumain concentration and severity score were tested by a linear regression model. Moreover, CAP patients were allocated into three subgroups in terms of the concentrations of serum legumain: tertile (T) 1, serum legumain <1.03 ng/mL; T2, 1.03 ng/mL<serum legumain <2.05 ng/mL; and T3, serum legumain>2.05 ng/mL. Therefore, the associations of serum legumain with prognostic outcomes were assessed by a logistic regression model. The predictive efficiencies of different parameters were determined by receiver operating characteristic (ROC) curves. *P* value*s* <0.05 were considered significantly different.

## Results

### Basic information

All 293 CAP patients were enrolled, and their basic information is shown in Table 1. The average age and systolic pressure gradually increased in CAP patients with increased serum legumain. There were 49 (50.0%) males in the T1 and T2 groups and 50 (51.5%) males in the T3 group. In addition, the incidences of hypertension and cerebral infarction were elevated in CAP patients with increased concentrations of circulatory legumain. There were no differences in smoking status, diabetes mellitus status, coronary heart disease status or bronchitis among the three subgroups. There were no differences in white blood cells (WBCs), neutrophils, lymphocytes, monocytes, alanine aminotransferase (ALT), aspartate aminotransferase (AST), uric acid, creatine kinase, creatine kinase isoenzyme, cardiac troponin I, or lactate dehydrogenase among CAP patients with different concentrations of serum legumain. However, dramatic differences in platelet, urea nitrogen, creatinine, myoglobin, procalcitonin, D-dimer, C-reactive protein (CRP), and interleukin-6 (IL-6) levels were detected in the three subgroups (Table 1).

### The concentration of serum legumain in CAP patients

**Figure 1:**
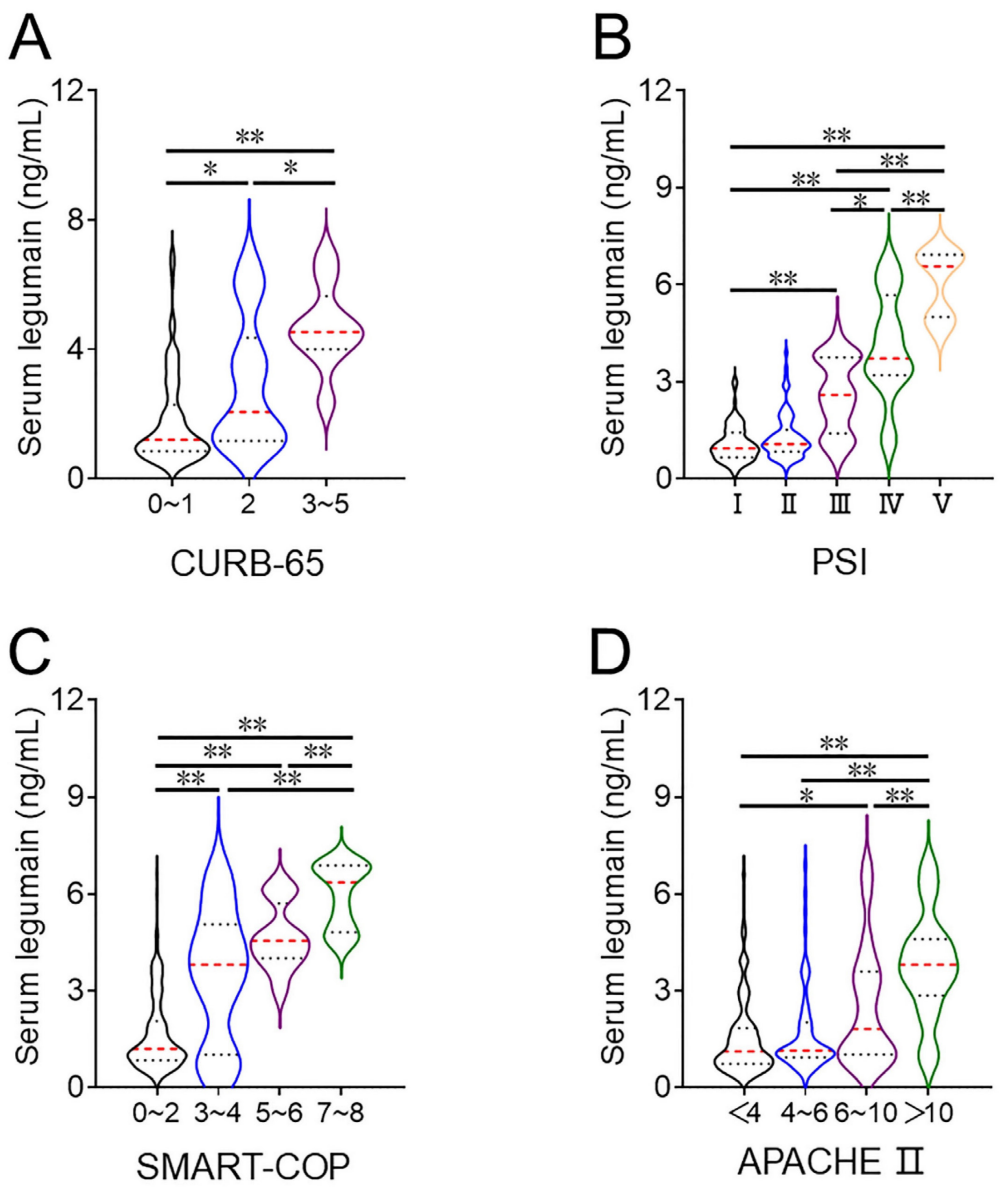
** Comparison of the serum legumain concentration in CAP patients with various disease severities.** The expression of legumain in serum samples was determined via ELISA. Differences in the serum legumain concentration were compared among CAP patients with different severity scores. (A) CURB-65. (B) PSI. (C) SMART-COP. (D) APACHE II score. *P<0.05, **P<0.01.

The concentration of serum legumain was detected. The concentration of serum legumain gradually increased in line with the CURB-65 score. In addition, the concentration of serum legumain was compared across different PSI score (Figure 1A). As shown in Figure 1B, the concentration of serum legumain was lowest in Grade Ⅰ patients and highest in Grade Ⅴ patients. According to the SMART-COP score, the expression of legumain increased with increasing severity (Figure 1C). Moreover, the increasing trend of the serum legumain concentration was similar to that of the APACHE II score (Figure 1D).

### Relationships of serum legumain with clinical characteristics in CAP patients

**Figure 2:**
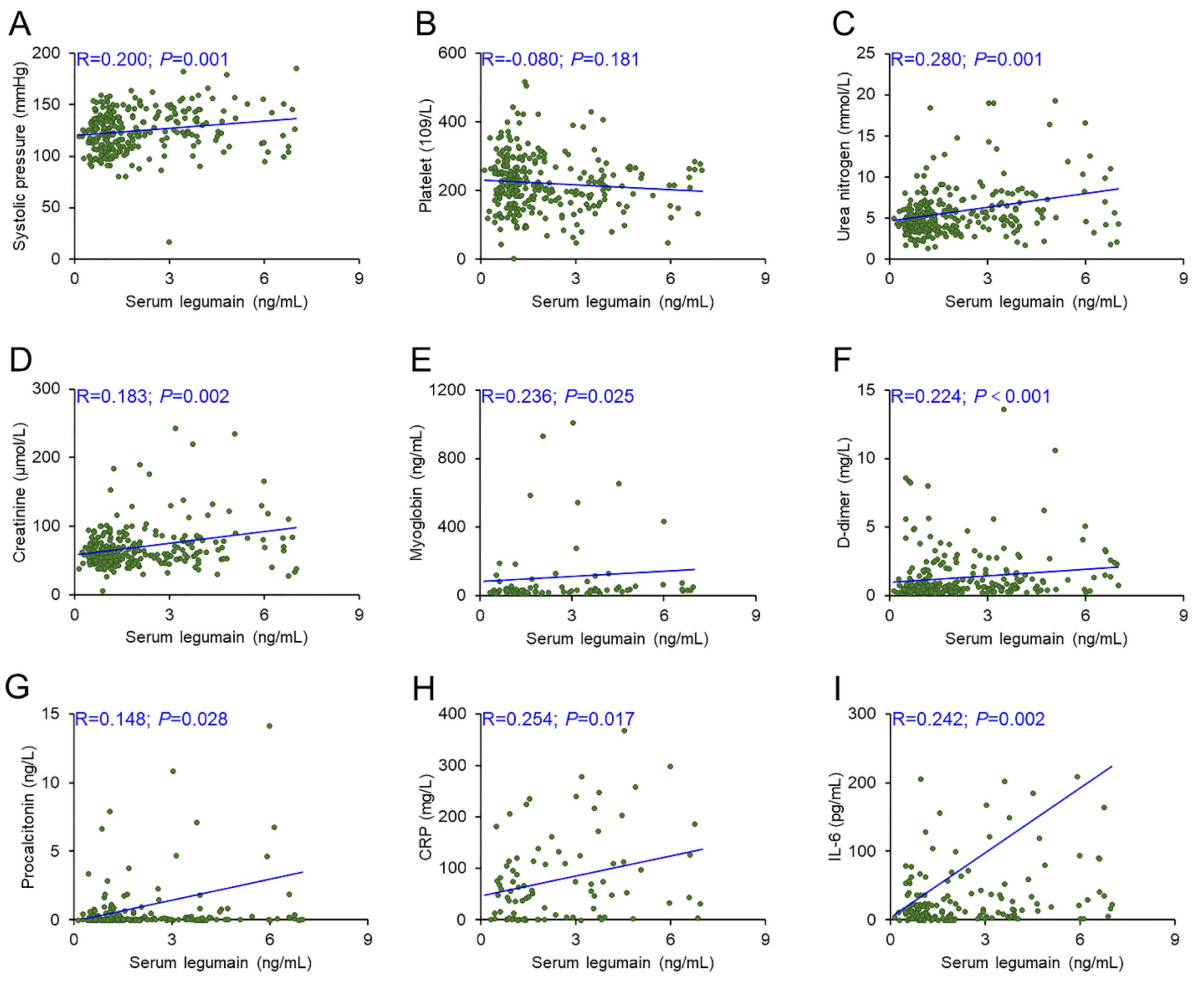
** Relationships of serum legumain with clinical indicators in CAP patients.** The correlations between the serum legumain concentration and clinical indicators were estimated through the Spearman rank correlation coefficient. (A) Correlation between serum legumain and systolic blood pressure. (B) Correlation between the serum legumain concentration and platelet count. (C) Correlation between serum legumain and urea nitrogen. (D) Correlation between serum legumain and creatinine. (E) Correlation between serum legumain and myoglobin. (F) Correlation between the serum legumain concentration and D-dimer level. (G) Correlation between the serum legumain level and the procalcitonin level. (H) Correlation between the serum legumain concentration and the CRP level. (I) Correlation between serum legumain and IL-6. The R values represent the correlation coefficient.

There were obvious differences in systolic pression, platelet count, urea nitrogen, creatinine, myoglobin, D-dimer, procalcitonin, CRP, and IL-6 among the three groups of CAP patients. The relationships between the serum legumain concentration and clinical characteristics were subsequently assessed via the Spearman correlation coefficient. The serum legumain concentration was positively associated with systolic pressure (R=0.200; *P*=0.001), urea nitrogen (R=0.280; *P*=0.001), creatinine (R=0.183; *P*=0.002), myoglobin (R=0.236; *P*=0.025), D-dimer (R=0.224; *P*<0.001), procalcitonin (R=0.148; *P*=0.028), CRP (R=0.254; *P*=0.017), and IL-6 (R=0.242; *P*=0.002) (Figure 2).

### Correlations of serum legumain with severity scores in CAP patients

A multivariate linear regression model revealed that each 1 ng/mL increase in the serum concentration of legumain was associated with an elevation of 0.059 (95% CI: 0.007~0.111) in the CURB-65 score, 8.504 (95% CI: 7.426~9.581) in the PSI score, 0.770 (95% CI: 0.659~0.882) in the SMART-COP score, and 0.350 (95% CI: 0.122~0.578) in the APACHE Ⅱ score (Table 2).

### Correlations of serum legumain with prognostic outcomes in CAP patients

**Figure 3:**
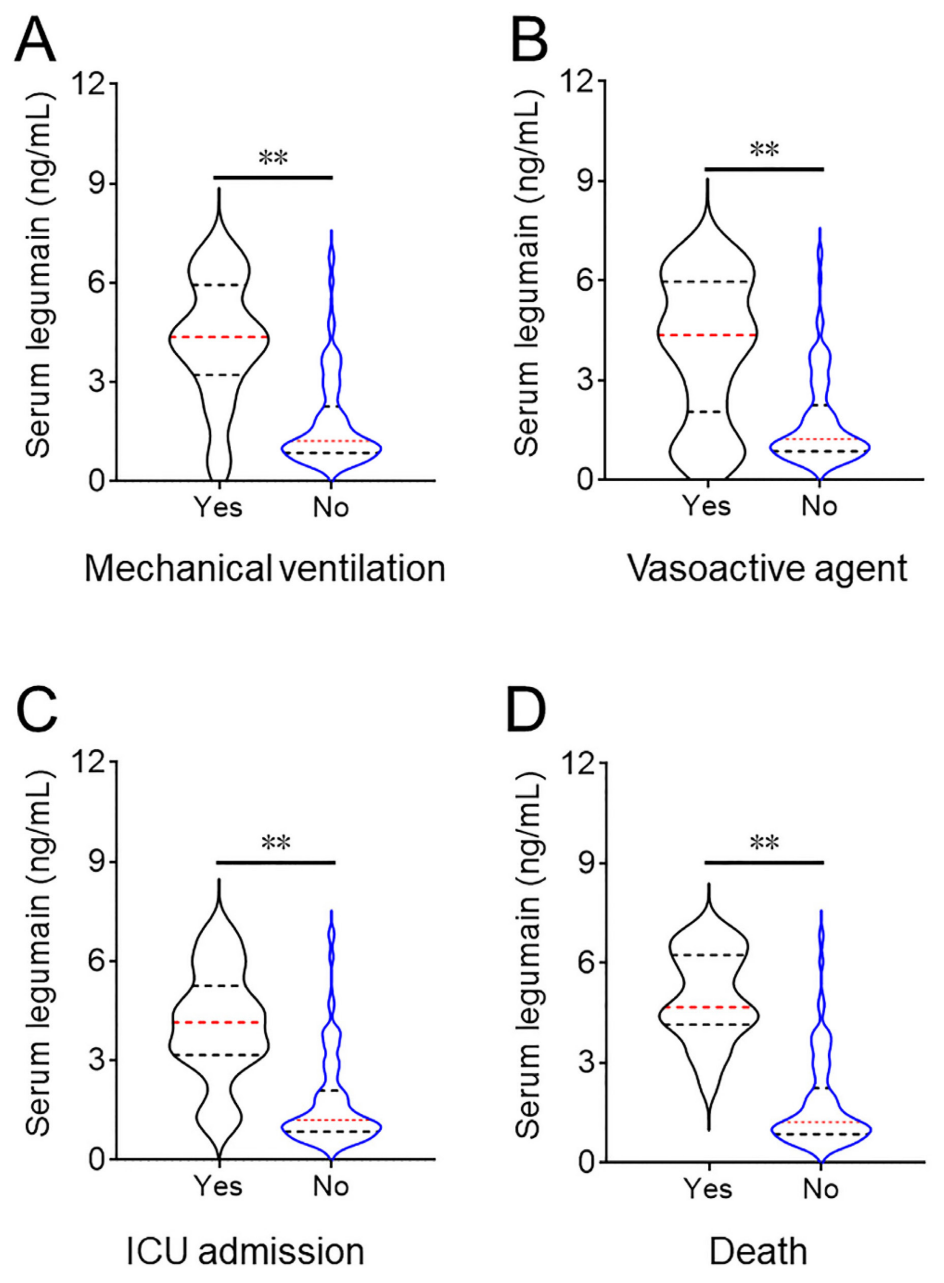
** Comparison of the serum legumain concentration in CAP patients with various prognoses.** The expression of serum legumain was determined using ELISA. Differences in the serum legumain concentration were compared in subjects with different prognoses. (A) Mechanical ventilation. (B) Vasoactive agent. (C) ICU admission. (D) Death. **P<0.01.

The serum legumain concentration at admission was dramatically increased in patients who underwent mechanical ventilation, were receiving vasoactive agents, were admitted to the ICU, and died during hospitalization (Figure 3). The relationships between the serum legumain concentration and prognostic outcomes were subsequently assessed. The χ^2^ test suggested that the number of patients with a poor prognosis increased with increasing serum legumain concentration (Table 3). Multivariate logistic regression analysis revealed that the relative risks (RRs) of mechanical ventilation, vasoactive agent use, and ICU admission were positively linked to the concentration of serum legumain in CAP patients at admission (Table 3).

### The predictive efficiency of serum legumain for prognosis in CAP patients

**Figure 4:**
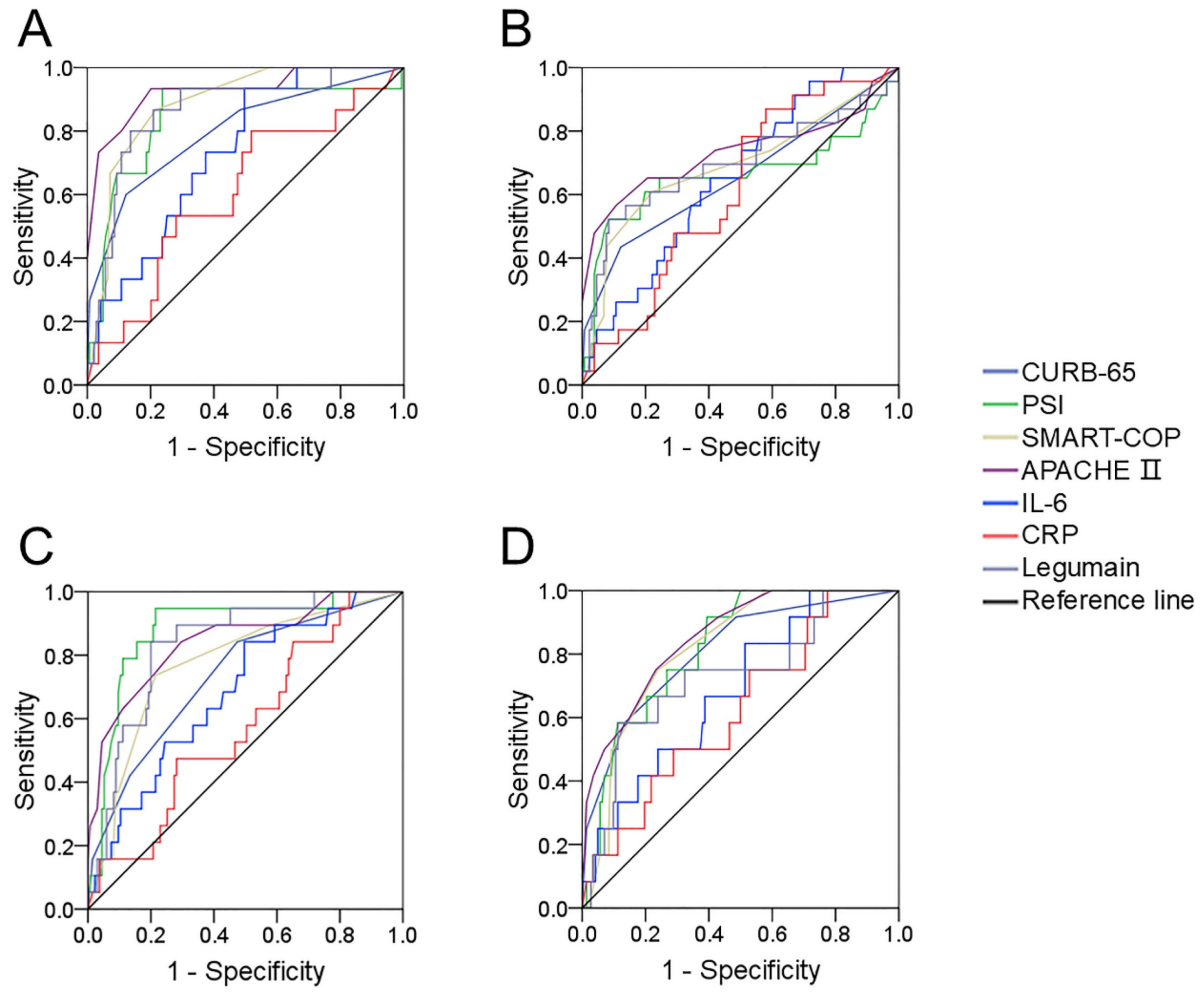
** The predictive power for various prognoses in CAP patients.** The predictabilities of various prognostic outcomes were assessed via receiver operating characteristic (ROC) curves. (A) Mechanical ventilation. (B) Vasoactive agent. (C) ICU admission. (D) Death.

The predictive efficiencies for prognostic outcomes were assessed by ROC curves. The areas under the ROC curve for mechanical ventilation were as follows: CURB-65, 0.812; PSI, 0.816; APACHE Ⅱ, 0.892; SMART-COP, 0.820; serum legumain, 0.844; IL-6, 0.734; and CRP, 0.610 (Figure 4A). In addition, the predictive powers for vasoactive agents were as follows: CURB-65, 0.677; PSI, 0.640; APACHE II, 0.691; SMART-COP, 0.660; serum legumain, 0.671; IL-6, 0.652; and CRP, 0.614 (Figure 4B). Moreover, the predictability for ICU admission was as follows: CURB-65, 0.797; PSI, 0.919; APACHE Ⅱ, 0.845; SMART-COP, 0.762; serum legumain, 0.839; IL-6, 0.685; and CRP, 0.577 (Figure 4C). Finally, the ability to predict death was as follows: CURB-65, 0.862; PSI, 0.838; APACHE II, 0.890; SMART-COP, 0.788; serum legumain, 0.799; IL-6, 0.685; and CRP, 0.621 (Figure 4D). Further analyses revealed no difference in the ability of circulatory legumain to predict the prognosis of patients with CAP according to severity score.

## Discussion

In this study, we first analysed the relationship between circulatory legumain and CAP and revealed that the serum legumain concentration was dramatically greater in severe patients than in mild patients. Additionally, the concentration of circulatory legumain was positively associated with severity scores. Moreover, the concentration of circulatory legumain on admission increased in patients with a poor prognosis during hospitalization. Higher circulatory legumain on admission elevated the risk of poor prognosis in hospitals. The predictive efficiency of circulatory legumain for poor prognostic outcomes was similar to that of severity scores.

Legumain, an endolysosomal cysteine protease, was initially found in common bean 10. There is increasing evidence suggesting that legumain is richly expressed in different types of human organs, such as the kidney, testis, placenta, heart, and midgut 26-29. Recently, researchers reported that legumain is also highly expressed in lung tissues 17-19. Moreover, an increasing number of studies have suggested that legumain is involved in a variety of inflammatory diseases, such as atherosclerosis, pancreatitis, atherosclerosis, and Alzheimer's disease 30-32. CAP is an inflammatory disease of the lung characterised by increased levels of inflammatory cytokines. We believe that legumain may play a partial role in CAP. The current research suggested that the legumain concentration was greater in severe patients than in mild patients. The linear regression model clearly revealed that legumain expression was positively related to the CURB-65, PSI, SMART-COP, and APACHE II scores. In addition, obvious correlations between serum legumain and clinical parameters, including renal function indicators and inflammatory cytokines, were found. These findings were in accordance with previous studies 33, 34. Collectively, these data strongly suggest that legumain may be involved in the pathophysiology of CAP.

This research suggested that legumain may be used for the diagnosis of CAP patients. Interestingly, a series of investigations have revealed that the expression level of legumain is related to prognosis in many diseases. Research has revealed that the expression of legumain is positively related to the risk of distant metastasis in gastric cancer patients 35. In addition, higher legumain expression increases the degree of malignancy in malignant human ovarian tumours 36. In addition, increased legumain is related to the malignant behaviour of uveal melanoma 37. Therefore, we considered that the serum legumain concentration could be used as a negative prognostic indicator for CAP. We found that the concentration of serum legumain was elevated in CAP patients with a poor prognosis during hospitalization. Additionally, higher serum levels of legumain are associated with a poor prognosis during hospitalization. The ability of the serum legumain concentration to predict a poor prognosis was similar to that of the severity score. In addition, the predictive efficiencies of serum legumain and inflammatory cytokines for poor prognosis were compared. These results suggested that the ability of serum legumain to predict a poor prognosis was greater than that of inflammatory cytokines. Therefore, these data suggest that the concentration of serum legumain may be a better prognostic factor for the adverse progression of CAP.

Serum legumain is only one serum biomarker that is convenient to detect and quick to acquire in patients. Although CAP severity scores have great advantages and potential for estimating the severity and prognosis among CAP patients, CAP severity scores consist of many indices and require more time to obtain. This means that it is easier to determine the concentration of serum legumain than to determine the CAP severity score. Moreover, the predictive power of serum legumain for a poor prognosis is greater than that of common routine blood indicators and inflammatory cytokines. Moreover, CAP patients with higher levels of serum legumain at admission have greater risks of mechanical ventilation, vasoactive agent usage, and ICU admission during hospitalization. These findings suggest that clinicians should pay more attention to those cases that may have poor prognostic outcomes in the future and receive timely treatment, such as respiratory support and drug intervention, to reduce the risk of mortality. Therefore, this method may be useful for measuring the level of serum legumain in CAP patients. However, the current research is no longer an observational study. If serum legumain detection is to be applied in clinical practice, there is still a long way to go.

We first revealed the relationship between serum legumain expression and CAP. We must recognise several limitations in this research. First, all participants were from two hospitals, and the sample size was relatively small. These defects may result in biases or overall overestimated associations. Therefore, a multicentre investigation with a larger sample size is needed in the future. Second, only circulatory legumain was measured. Lung tissues and bronchoalveolar lavage fluid cannot be obtained from CAP patients. Therefore, the local expression of legumain cannot be measured in lung tissues or bronchoalveolar lavage fluid. Third, the present research was only an observational study, and the mechanism of legumain elevation in CAP patients remains to be clarified. Previous studies have indicated that ferroptosis occurs during the progression of pneumonia 38, 39. The expression of legumain is increased in acute kidney injury, and increased legumain expression can aggravate the degradation of GPX4 and evoke ferroptosis during acute kidney injury 15. Moreover, legumain is overexpressed in macrophages in inflammatory diseases. Proinflammatory macrophages are activated in the lung tissues of patients with viral pneumonia and secrete legumain 40, 41. Thus, we hypothesise that ferroptosis and macrophage aviation, or other physiological processes, may be involved in the progression of CAP and mediate the increase in legumain. In vivo and in vitro studies may help illuminate the mechanism of legumain upregulation in CAP patients.

## Conclusion

Taken together, this study is the first to explore the relationship between the serum legumain concentration and CAP and the clinical significance of the serum legumain concentration on the basis of a prospective cohort study. This study revealed that the serum legumain concentration is elevated in severe patients. The serum legumain concentration is strongly positively related to severity and adverse prognosis, suggesting that legumain may be involved in the physiopathological processes of CAP. Therefore, increased serum legumain is a poor factor for severity and prognosis and can guide clinical decisions for CAP patients. The serum legumain concentration might be used as a possible biomarker and a molecular goal for CAP therapy in the clinic.

## Figures and Tables

**Table 1:** Basic information.

Characteristics	Serum legumain (ng/mL)			*P*
	T1 (<1.03)	T2 (1.03~2.05)	T3 (>2.05)	
N	98	98	97	
Age, years	47.7±1.95	55.7±1.64	70.7±1.25	**<0.001**
Male, n (%)	49 (50.0)	49 (50.0)	50 (51.5)	0.967
Body mass index	22.7±0.43	22.0±0.45	22.2±0.51	0.573
Systolic pressure (mmHg)	120.6±1.74	122.5±1.89	131.2±2.47	**0.001**
Diastolic pressure (mmHg)	74.3±0.99	76.0±1.16	76.7±1.41	0.351
Smoker, n (%)	16 (16.3)	17 (17.3)	19 (19.6)	0.826
Hypertension, n (%)	17 (17.3)	19 (19.4)	40 (41.2)	**<0.001**
Diabetes mellitus, n (%)	5 (5.1)	9 (9.2)	9 (9.3)	0.483
Cerebral infarction, n (%)	5 (5.1)	2 (2.0)	15 (15.5)	**0.001**
Coronary heart disease, n (%)	6 (6.1)	6 (6.1)	5 (5.2)	1.000
Bronchitis, n (%)	2 (2.0)	1 (1.0)	2 (2.1)	0.874
White blood cell (10^9^/L)	7.7±0.40	7.2±0.38	8.0±0.50	0.438
Neutrophil (10^9^/L)	6.3±0.89	5.2±0.35	6.9±0.98	0.277
Lymphocyte (10^9^/L)	1.6±0.12	1.4±0.10	1.3±0.12	0.215
Monocyte (10^9^/L)	0.6±0.03	0.6±0.04	0.6±0.03	0.816
Platelet (10^9^/L)	229.3±7.6	229.5±10.5	202.3±7.83	**0.045**
Alanine aminotransferase (U/L)	31.0±2.88	29.6±4.35	31.3±3.73	0.948
Aspartate aminotransferase (U/L)	28.1±2.28	32.0±5.74	39.7±6.35	0.258
Uric acid (μmol/L)	265.8±9.10	284.5±12.36	293.6±13.25	0.229
Urea nitrogen (mmol/L)	4.6±0.15	5.5±0.27	7.2±0.56	**<0.001**
Creatinine (μmol/L)	58.0 (48.0, 67.8)	60.0 (48.0, 71.0)	68.0 (52.0, 86.0)	**0.002**
Creatine kinase (U/L)	58.5 (40.8, 95.8)	54.0 (38.0, 88.0)	68.0 (46.5, 106.5)	0.495
Creatine kinase isoenzyme (U/L)	13.0 (10.0, 17.0)	11.0 (9.0, 14.0)	12.5 (8.0, 17.0)	0.272
Myoglobin (ng/mL)	30.4 (19.1, 42.1)	22.4 (17.7, 35.9)	33.5 (25.8, 82.5)	**0.029**
Cardiac troponin I (ng/mL)	0.01 (0, 0.10)	0.01 (0, 0.10)	0.02 (0.01, 0.64)	0.279
Lactate dehydrogenase (U/L)	210.4±9.00	217.3±16.30	251.2±25.74	0.346
Procalcitonin (ng/L)	0.05 (0.02, 0.14)	0.06 (0.02, 0.19)	0.10 (0.04, 0.56)	**0.021**
D-dimer (mg/L)	1.2±0.23	1.1±0.13	1.6±0.22	**<0.001**
C-reactive protein (mg/L)	46.0 (2.7, 86.4)	40.0 (1.9, 85.0)	74.9 (19.6, 176.7)	**0.025**
Interleukin-6 (pg/mL)	11.6 (3.4, 22.6)	6.4 (1.9, 23.8)	27.8 (10.2, 90.2)	**0.001**

Data in bold denote statistically significant results.

**Table 2:** Correlations between serum legumain and CAP severity scores.

Variables	Estimated changes by serum legumain β (95% CI)
N	293
CURB-65	**0.059 (0.007, 0.111)**
PSI	**8.504 (7.426, 9.581)**
SMART-COP	**0.770 (0.659, 0.882)**
APACHE Ⅱ	**0.350 (0.122, 0.578)**

Models were adjusted for age, hypertension, diabetes mellitus, cerebral infarction, coronary heart disease, and bronchitis. Data in bold denote statistically significant results.

**Table 3:** Correlations between serum legumain and prognostic outcomes.

Variables	Serum legumain (ng/mL)			*P* trend
	T1 (<1.03)	T2 (1.03~2.05)	T3 (>2.05)	
N	98	98	97	
Mechanical ventilation				
N, (%)	2 (2.0)	1 (1.0)	18 (18.6)	**<0.001**
RR	Ref (1.0)	0.452 (0.039, 5.180)	**9.839 (1.718, 56.342)**	**0.001**
Vasoactive agent				
N, (%)	9 (9.2)	5 (5.1)	20 (20.6)	**0.003**
RR (Model 2)	Ref (1.0)	0.562 (0.175, 1.806)	**3.686 (1.213, 11.199)**	**0.018**
ICU admission				
N, (%)	1 (1.0)	4 (4.1)	24 (24.7)	**<0.001**
RR	Ref (1.0)	2.580 (0.270, 24.679)	**11.446 (1.404, 93.327)**	**0.001**
Death				
N, (%)	3 (3.1)	1 (1.0)	12 (12.4)	**0.001**
RR	Ref (1.0)	0.230 (0.023, 2.330)	2.226 (0.504, 9.822)	0.130

RR: Relative risk. Models were adjusted for age, hypertension, diabetes mellitus, cerebral infarction, coronary heart disease, and bronchitis. Data in bold denote statistically significant results.
